# On‐Site Melanoma Diagnosis Utilizing a Swellable Microneedle‐Assisted Skin Interstitial Fluid Sampling and a Microfluidic Particle Dam for Visual Quantification of S100A1

**DOI:** 10.1002/advs.202306188

**Published:** 2024-02-28

**Authors:** Gaobo Wang, Yuyue Zhang, Hoi Kwan Kwong, Mengjia Zheng, Jianpeng Wu, Chenyu Cui, Kannie W. Y. Chan, Chenjie Xu, Ting‐Hsuan Chen

**Affiliations:** ^1^ Department of Biomedical Engineering City University of Hong Kong 83 Tat Chee Avenue Kowloon Tong Hong Kong SAR 999077 China; ^2^ City University of Hong Kong Shenzhen Research Institute 8 Yuexing 1st Road, Shenzhen Hi‐Tech Industrial Park, Nanshan District Shenzhen 518057 China; ^3^ Hong Kong Centre for Cerebro‐Cardiovascular Health Engineering Rm 1115‐1119, Building 19W, 19 Science Park West Avenue Hong Kong SAR 999077 China

**Keywords:** interstitial fluid, malignant melanoma, microfluidics, S100A1, swellable microneedle

## Abstract

Malignant melanoma (MM) is the most aggressive form of skin cancer. The delay in treatment will induce metastasis, resulting in a poor prognosis and even death. Here, a two‐step strategy for on‐site diagnosis of MM is developed based on the extraction and direct visual quantification of S100A1, a biomarker for melanoma. First, a swellable microneedle is utilized to extract S100A1 in skin interstitial fluid (ISF) with minimal invasion. After elution, antibody‐conjugated magnetic microparticles (MMPs) and polystyrene microparticles (PMPs) are introduced. A high expression level of S100A1 gives rise to a robust binding between MMPs and PMPs and reduces the number of free PMPs. By loading the reacted solution into the device with a microfluidic particle dam, the quantity of free PMPs after magnetic separation is displayed with their accumulation length inversely proportional to S100A1 levels. A limit of detection of 18.7 ng mL^−1^ for S100A1 is achieved. The animal experiment indicates that ISF‐based S100A1 quantification using the proposed strategy exhibits a significantly higher sensitivity compared with conventional serum‐based detection. In addition, the result is highly comparable with the gold standard enzyme‐linked immunosorbent assay based on Lin's concordance correlation coefficient, suggesting the high practicality for routine monitoring of melanoma.

## Graphical abstract

A swellable hydrogel microneedle patch is employed for on‐site sampling of S100A1 in skin interstitial fluid. After mixing with antibody‐conjugated magnetic microparticles (MMPs) and polystyrene microparticles (PMPs), the expression level of S100A1 is displayed with a visual bar formed by the accumulated PMPs using a self‐powered microfluidic device with a magnetic separator and a microparticle dam.

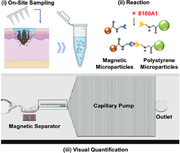

## 1 Introduction

The world has increased by ≈325 000 new malignant melanoma (MM) cases in 2020 with the consequences of ≈57000 deaths, and is expected to have 510 000 new cases and 96 000 deaths by 2040.^[^ ^1^ ^]^ As skin cancer originating from skin melanocytes with a high death rate, MM has caused 75% of its deaths although its incidence rate only accounts for 4% of the total incidence of skin cancers.^[^ ^2^ ^]^ Due to the rapid metastasis, the majority of patients are diagnosed at the late stage with poor prognosis, to which the conventional treatment methods including radiotherapy, chemotherapy and immunotherapy are not effective.^[^ ^3^ ^]^ As such, a preventive measurement by taking a regular skin examination is of great significance for improving the prognosis and elevating the quality of life.

Dermoscopy is primarily the first option taken to observe the microscopic substructures under the epidermis through 2D projections of the skin tissue structure at different depths.^[^ ^4^ ^]^ For instance, MetaOptima's MoleScope is a skin magnifying glass that works with a mobile phone to allow daily self‐inspections by end users.^[^ ^5^ ^]^ However, MM at its relatively early stage is not easy to be discriminated since it shares many clinical features with benign moles, making such non‐invasive dermoscopy‐based diagnosis less effective.

In this regard, patients with dermoscopy observation indicating a high risk of MM will be additionally asked to conduct the surgical excision (tissue biopsy) followed by immunohistochemical staining for markers including S100, HMB45 and Melan A etc. for further evaluation.^[^ ^6^ ^]^ However, tissue biopsy is invasive, painful, and likely to leave a scar on the patient's skin. As such, such incisional surgery is usually conducted in the case of metastatic cases to minimize further severe consequences, making it impractical for routine screening.

Instead, liquid biopsies including serum/plasma, urine and other body fluids are much preferred for tumor diagnosis and tracking of biomarkers, thanks to its significantly quicker turnaround for test results.^[^ ^7^ ^]^ There are MM‐related biomarkers circulating in blood including S100 proteins family, miRNA, and exosomes etc.^[^ ^8^ ^]^ Notably, S100A1, as a member of the S100 family of Ca^2+^‐binding proteins, exists as a dimer of two identical monomers and each monomer contains 94 amino acids, with a molecular weight of 10,546 Da.^[^ ^9^ ^]^ The serum S100A1 collected from patients who have been diagnosed with MM has exhibited higher expression levels compared with the non‐melanoma cases.^[^ ^10^, ^11^, ^12^ ^]^ However, the overexpression of serum S100A1 may to be related to other diseases such as breast cancer, cardiovascular disease and ovarian cancer etc., making the serum‐based S100A1 detection less specific.^[^ ^13^, ^14^, ^15^, ^16^ ^]^ In addition, while the enzyme‐linked immunosorbent assay (ELISA) measurement has been regarded as the gold standard for the determination of S100A1, the tedious procedure and the requirement for a bench‐top microplate reader for signal quantification make it unsuitable for the general public.

**Figure 1:**
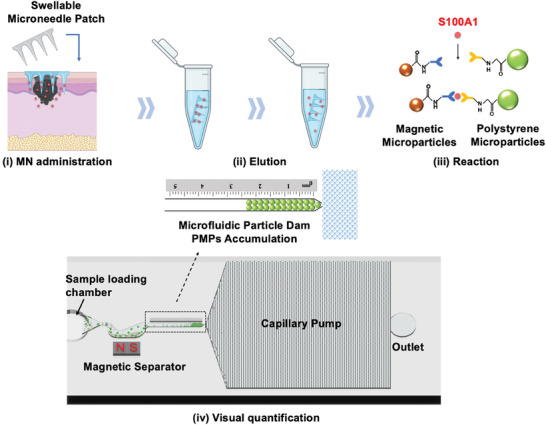
Illustration of a swellable microneedle patch and a self‐powered microfluidic device for melanoma diagnosis. i) Extraction of ISF with the administration of a swellable microneedle patch on the skin. ii) Elution in a centrifuge tube. iii) Reaction between S100A1 and antibody‐conjugated magnetic microparticles (MMPs) and polystyrene microparticles (PMPs). iv) Visual quantification of S100A1 using a microfluidic device with a microfluidic particle dam. With the high concentration of S100A1, MMPs and PMPs simultaneously bind to S100A1 to form the “MMPs‐S100A1‐PMPs” structure, which lessens the number of free PMPs escaping from a magnetic separator, resulting in a short PMP accumulation length that can be visually quantified without an additional instrument. With the lower concentration of S100A1, however, a longer PMP accumulation length is observed due to the insufficient binding between MMPs and PMPs.

Comparatively, the skin interstitial fluid (ISF) in the epidermis or upper dermis has been known to possess protein diversity comparable to that in blood.^[^ ^17^ ^]^ Microneedles, with a length of less than 1000 µm, are developed for ISF extraction by penetrating through the stratum corneum of skin without touching the nerve tissues. Importantly, with direct administration of microneedle onto skin‐related diseases (e.g., Melanoma and Psoriasis), the microneedle‐assisted ISF extraction offers significant reliability and practicality owing to the easy‐access property.^[^ ^18^, ^19^, ^20^ ^]^ Moreover, to ease the downstream analysis, the integration of microneedle‐assisted sampling with microfluidic technology for household health monitoring has been investigated.^[^ ^21^, ^22^, ^23^, ^24^ ^]^ While the microneedle‐based ISF extraction of biomarkers (e.g., S100B, Tyrosinase and Lactate) for melanoma diagnosis has been established recently, the detections are based on fluorescence or colorimetric sensing, which are either impractical or non‐quantitative.^[^ ^25^, ^26^, ^27^ ^]^ In this work, we proposed a minimally invasive, on‐site sampling of S100A1 by utilizing a swellable microneedle patch (microneedle), followed by a direct visual quantification of S100A1 based on a self‐powered device with a microfluidic particle dam (**Figure** **1**). After sampling, the extracted ISF is eluted and mixed with magnetic microparticles (MMPs) and polystyrene microparticles (PMPs) immobilized with monoclonal antibodies. S100A1 existing in ISF with high concentration leads to robust binding between MMPs and PMPs, forming the “MMPs‐S100A1‐PMPs” sandwich structure and resulting in a reduced number of free PMPs. To quantify the free PMPs, a microfluidic device consisting of a sample loading chamber, a magnetic separator, a trapping channel with a microparticle dam and a capillary pump is developed. The mixed solution is first loaded into the sample loading chamber and flows into the microchannel. The “MMPs‐S100A1‐PMPs” sandwich structure is trapped in the bottom of the magnetic separator, while free PMPs continuously flow into the trapping channel and accumulate at the microparticle dam. Thus, the level of S100A1 is reflected by the PMP accumulation length, which can be directly visualized without additional instruments. Based on the established strategy, the limit of detection was calculated to be 18.7 ng mL^−1^ utilizing a skin model containing S100A1 standards. Furthermore, in vivo microneedle‐assisted sampling of S100A1 was conducted using the mice animal model with different tumor sizes. The results showed that the expression level of S100A1 is elevated with the increased tumor sizes according to both ELISA and microfluidic chip measurement. As a comparison, conventional detection methods including serum‐based detection and immunohistochemistry staining for S100A1 quantification were also conducted but no correlation was observed, demonstrating the high sensitivity of interstitial fluid‐based detection. In addition, on the basis of Lin's concordance correlation coefficient, our device provides quantitative results highly comparable to that from the conventional gold standard ELISA measurement. It is believed that the proposed strategy by combining the swellable microneedle patch and microfluidic particle dam has great potential for routine skin inspection and diagnosis of melanoma in the future.

## 2 Results and Discussion

### 2.1 Optimization of Hyaluronic Acid (HA) with Different Molecular Weights

**Figure 2:**
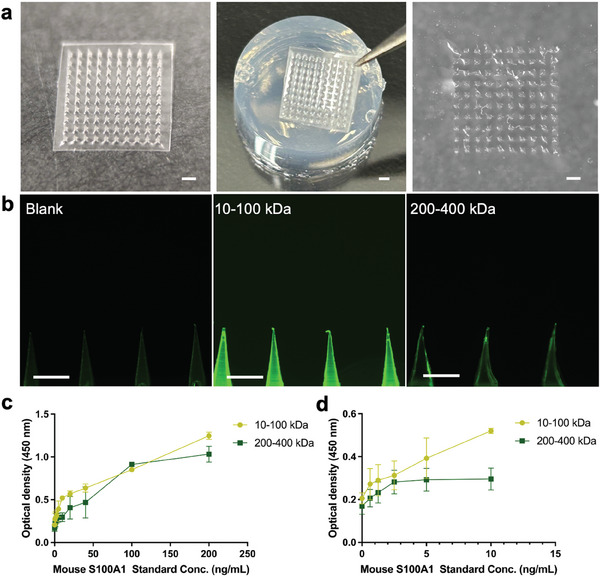
Optimization of hydrogel‐based swellable microneedle for extraction of macromolecules. a) Optical image of microneedles (left), the agarose gel before (middle) and after the insertion (right). Scale bar: 1 mm. b) Demonstration of FITC‐dextran (molecular weight: 15 kDa) extraction from the skin model. The control group (left, agarose gel without FITC‐dextran), the 10–100 kDa HA fabricated microneedle group (middle) and the 200–400 kDa HA fabricated microneedle group (right). Scale bar: 500 µm. c) Optical density at wavelength 450 nm to measure the extracted S100A1 concentration in skin model (0, 0.625, 1.25, 2.5, 5, 10, 20, 40, 100, and 200 ng mL^−1^) using HA fabricated microneedles (n = 3). d) Subset data of Figure 2c where the concentration of S100A1 ranges from 0 – 10 ng m^−1^L (n = 3).

We have previously developed a hydrogel‐based swellable microneedle patch based on methacrylated hyaluronic acid (MeHA) that is capable of absorbing protein biomarkers after skin penetration in less than 1 min (**Figure** **2**a‐b; Figure S1a, Supporting Information).^[^ ^28^, ^29^ ^]^ The synthesized MeHA was first characterized using ^1^H NMR (Figure S1b, Supporting Information), which suggests the successful modification of HA with methacrylate and thus the MeHA could be further cross‐linked. The mechanical strength of microneedles made by different molecular weights of HA (10–100 and 200–400 kDa) measured by compression test exhibited similar mechanical properties (Figure S1c, Supporting Information). The force load reaches 1 N per needle, indicating that the microneedle could penetrate the skin layer under the thumb press without deformation. To optimize the molecular weights of HA used for the synthesized MeHA for the fabrication of microneedles, we then visualized the extraction ability for macromolecules based on the microneedles fabricated with MeHA, which was synthesized from 10–100 kDa and 200–400 kDa HA, respectively. The FITC‐dextran (Molecular weight: 15 kDa) was prepared in a skin model using 1.3 wt.% agarose hydrogel with an elastic modulus of ≈20 kPa.^[^ ^30^, ^31^ ^]^ After penetration into the skin model, the punched holes could be observed clearly on the surface (Figure 2a). Both microneedles fabricated with 10–100 kDa and 200–400 kDa HA synthesized MeHA exhibited successful extraction of FITC‐dextran in comparison with the control group (agarose gel without the addition of FITC‐dextran (Figure 2b). Clearly, the 10–100 kDa HA fabricated microneedles indicated a higher fluorescence intensity, demonstrating a better extraction performance. To further compare the extraction capability for macromolecules like protein biomarkers, mouse S100A1 protein with different concentrations was dissolved in the skin model, extracted using the microneedles and analyzed using ELISA. In general, we observed a linear increase in absorbance value with an elevated concentration of S100A1 in the skin model (Figure 2c,d), especially for microneedles fabricated from 10–100 kDa HA. Therefore, the 10–100 kDa HA was selected for the synthesis of MeHA and microneedles fabrication.

### 2.2 Detection of Mouse S100A1 Standards on the Microchips

**Figure 3:**
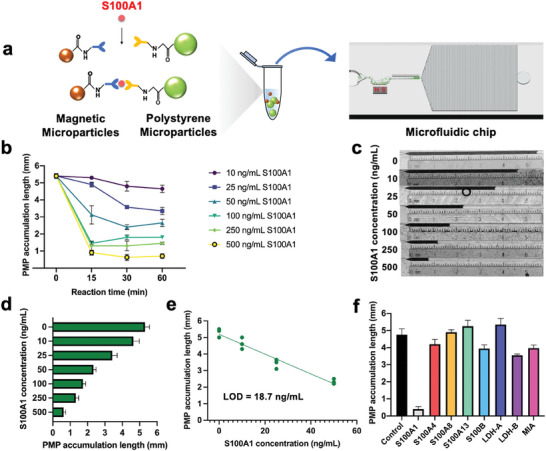
Detection of mouse S100A1 standards on microchip. a) Schematic illustration of S100A1 detection using the microfluidic chip. b) Optimization of reaction time (n = 3). c) Optical image of accumulated PMPs. d) PMP accumulation length versus with respect to S100A1 concentrations (n = 3). e) Linear regression with S100A1 concentration ranging from 0 to 50 ng mL^−1^. f) Selectivity against potential interfering factors (n = 3). The measured PMP accumulation length showed that only S100A1 (500 ng mL^−1^) shortened the PMP accumulation while other interfering biomarkers with higher concentrations (S100A4, 1 µg mL^−1^; S100A8, 250 µg mL^−1^; S100A13, 1 µg mL^−1^; S100B, 1 mg mL^−1^; LDH‐A, 10 µg mL^−1^; LDH‐B, 10 µg mL^−1^; MIA, 1 mg mL^−1^) did not cause any robust binding between MMPs and PMPs.

We next investigated the performance of microchips for the detection of mouse S100A1 protein. The standards were prepared by diluting mouse S100A1 in a buffered solution. As noted above, the “MMPs‐S100A1‐PMPs” sandwich structure is formed with the presence of S100A1 using the antibodies conjugated microparticles (PMPs‐Cap and MMPs‐Det). With the absence of S100A1, however, PMPs are disconnected from MMPs, resulting in an increased number of free PMPs. Such differences were distinguishable by loading the particle solution into the microchip consisting of a sample loading chamber, a magnetic separator, a trapping channel with a nozzle (narrowest width: 8 µm) and a capillary pump (Figure 1). When the solution passes through the magnetic separator, the “MMPs‐S100A1‐PMPs” sandwich structure and MMPs are trapped due to the magnetic force while free PMPs flow into the trapping channel until being blocked at the nozzle, forming a bar of trapped PMPs that can be visualized by the naked eye (**Figure** **3**a). To maximize the trapping efficiency, we designed the magnetic separator in the shape of a human stomach to decelerate the flow and guide the stream toward the magnets. The magnetic field intensity across the stomach‐shape magnetic separator applied by the attached neodymium magnet (2.6 mm × 1.8 mm × 1.5 mm) was calculated ranging from 2 to 16 mT mm^−1^ based on the magnetization value of 7.84 × 10^4^ A m^−1^ measured by a gaussmeter (Model VGM, AlphaLab Inc., USA).^[^ ^32^ ^]^ For microparticle modification, the amount of antibody was first optimized using flow cytometry. For PMPs modification (15.3 µm in diameter, 5.146 × 10^7^ microspheres mL^−1^), the fluorescence intensity was enhanced with the increasing of capture antibody amount modified per mg PMPs, reaching a plateau at 75 µg capture antibody per mg PMPs (Figure S2a, Supporting Information). For MMPs modification (0.797 µm diameter, 1.725 × 10^11^ microspheres mL^−1^), the ratio was optimized to be 300 µg detection antibody per mg MMPs (Figure S2b, Supporting Information). Based on this preliminary optimization, we next further optimized antibody conjugation and the number of microparticles using a microfluidic chip (Figure S2c–e, Supporting Information). Finally, 50 µg capture antibody per mg PMPs, 2.5 mg mL^−1^ MMPs and 200 µg detection antibody per mg MMPs were selected. Next, the reaction time for MMPs, PMPs and S100A1 was optimized to shorten the turnaround time (Figure 3b). While the PMP accumulation length increased with varying degrees in some groups (50, 100, 250, and 500 ng mL^−1^) at 15 min due to the broken of the non‐covalent bonding of MMP‐S100A1‐PMP, sufficient reaction was achieved within 30 min. Therefore, 30 min was used for the following experiment. Next, a series of concentrations of S100A1 standards (0, 10, 25, 50, 100, 250, and 500 ng mL^−1^) was used to explore the limit of detection of S100A1. The results showed that the PMP accumulation length is inversely proportional to the S100A1 concentration (Figure 3c). Using the linear interval from the blank sample (0 − 50 ng mL^−1^), the linear regression equation was determined as $y\;=\;-0.061x+5.249\pm 0.4895\sqrt{\frac{1}{3}+\frac{1}{12}+\frac{{\left(x-21.25\right)}^{2}}{4256.25}}\;$(R^2^ = 0.948), where *x* represents the S100A1 concentration and the uncertainties of the intercept s_b0_ and slope s_b1_ are 0.1291 and 0.0045, respectively. Based on the linear regression, the limit of detection (LOD) was defined to be 18.7 ng mL^−1^ (Figure 3d,e). To test the tolerance against other interfering factors that may present in melanoma tissues, other potential biomarkers including S100A4, S100A8, S100A13, S100B, Lactate Dehydrogenase A (LDH‐A), Lactate Dehydrogenase B (LDH‐B) and Melanoma Inhibitory Activity (MIA) were selected. Different concentrations of interfering biomarkers were used for selectivity tests since we found that extremely high concentrations (larger than 1 µg mL^−1^) of S100A4 and S100A13 would still cause non‐specific bonding between MMPs and PMPs. Similar selection criteria of concentrations were used for other interfering factors. The results showed that the interferences did not cause any unintended connection between MMPs and PMPs even with concentrations much higher than that of S100A1, indicating the excellent selectivity of antibody‐conjugated microparticles for S100A1 detection (Figure 3f).

### 2.3 In Vitro Detection of S100A1 Extracted from Skin Model

**Figure 4:**
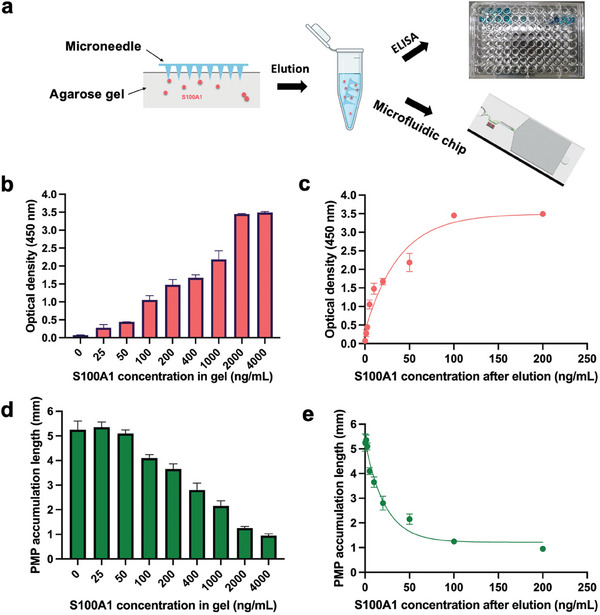
Standard curves for S100A1 extracted from skin model. a) Schematic illustration of S100A1 extraction using microneedle patch, followed by ELISA and microchip analyses. b) ELISA measurement for S100A1 in the skin model. c) Standard curve of ELISA measurement using non‐linear fitting d) PMP accumulation length in microfluidic chip against S100A1 concentrations in the skin model. e) Standard curve of microchip measurement using the non‐linear fitting. All experiments were repeated twice.

We next established the standard curve for S100A1 detection using ELISA and the microfluidic chip. A skin model was utilized containing a series of S100A1 concentrations (0, 25, 50, 100, 200, 400, 1000, 2000, and 4000 ng mL^−1^). The microneedles were weighed before and after extraction to determine the extracted volumes. After the administration of microneedles, the S100A1 was eluted in the dilution buffer overnight (**Figure** **4**a). Based on the extracted volumes, the volume of added sample dilution buffer was in accordance with the extracted volume to ensure a consistent 20 times dilution.^[^ ^29^ ^]^ Based on ELISA measurements, the optical density increased with the increase of S100A1 concentration in the skin model (Figure 4b). Using the dilution factor determined by the microneedle weight and the volume of the elution buffer, the non‐linear fitting curve between the S100A1 concentration after elution and the optical density was established to be $y\;=\;1.5405logx-0.071+0.727\sqrt{\frac{1}{2}+\frac{1}{16}+\frac{{\left(logx-26.79\right)}^{2}}{16017.85}}$ (R^2^ = 0.95) (Figure 4c; Statistical analysis, Supporting Information). Meanwhile, the same samples were measured using the microfluidic chip (Figure 4d). With the increasing concentration of S100A1, fewer free PMPs were accumulated in the trapping channel due to the robust binding between MMPs and PMPs, resulting in a short PMP accumulation length. The standard curve for on‐chip detection was thus determined to be $y\;=\;-2.122logx+5.687+0.679\sqrt{\frac{1}{2}+\frac{1}{16}+\frac{{\left(logx-26.79\right)}^{2}}{16017.85}}$ (R^2^ = 0.983) (Figure 4e; Statistical analysis, Supporting Information). In addition, the LOD of ELISA and microfluidic chip measurements for skin models were calculated to be 41.7 and 117.3 ng mL^−1^, respectively, based on the non‐central t‐distribution (Figure S3 and Equation S7, Supporting Information).

### 2.4 Biocompatibility of the Swellable Microneedles

**Figure 5:**
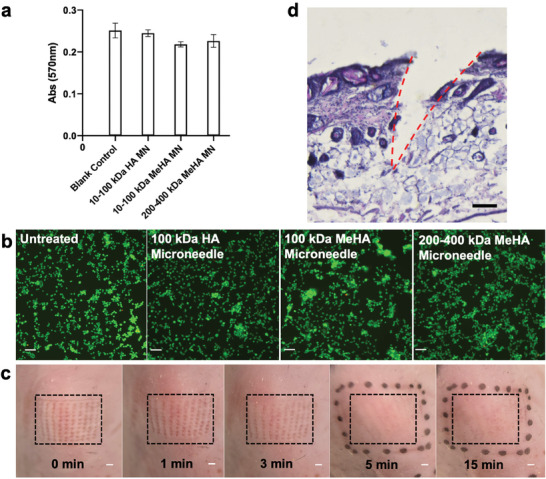
Biocompatibility of the swellable microneedles. a) The cell viability test via Alamar blue staining (n = 3). b) Images of live and dead assay of MSC viability treated with blank, 100 kDa HA, 100 kDa MeHA, and 200–400 kDa MeHA fabricated microneedles. Scale bar: 100 µm c) Optical images of mice skin before and 1, 3, 5, and 15 min after microneedle penetration. Scale bar: 1 mm d) The H&E staining of the mouse skin after microneedle penetration. Scale bar: 100 µm.

Before conducting the animal experiment, the potential side effect of the microneedles was first investigated (**Figure** **5**). The AlamarBlue cell viability assay indicated that there was no significant difference regarding the cell viability of skin fibroblasts after being treated with different microneedle soaking solutions (Figure 5a). A similar conclusion could also be drawn from the fluorescence images of the Live/Dead staining (Figure 5b). We then studied the microneedle penetration performance on mice skin (Figure 5c). After the removal of the microneedle patch, clear arrays of micropores could be observed, which became invisible in 15 min. Therefore, it could conclude that the sampling procedure of MeHA microneedle patch penetration would not bring significant damage or deformation to the skin, which would finally recover after removing the microneedle patch. From the H&E staining results (Figure 5d), the microneedle patch can penetrate the stratum corneum layer of mice skin and reach the dermis at a depth of ≈450 µm, which is sufficient to extract ISF for post‐analysis without touching nerves.

### 2.5 Immunohistology Staining of S100A1 in Animal Model

**Figure 6:**
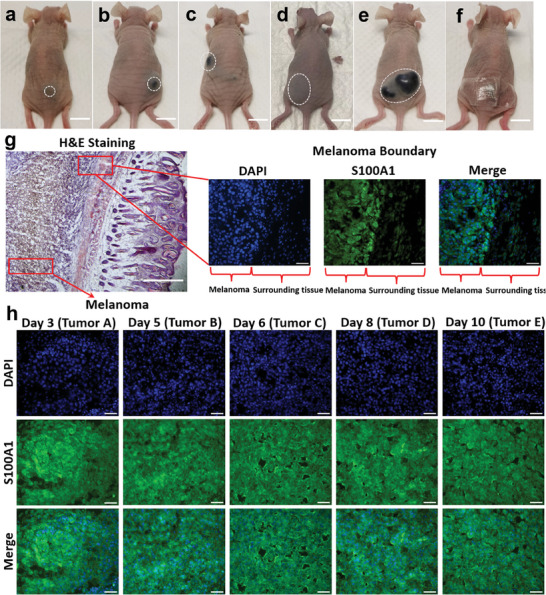
In vivo model of melanoma. a–e) Mice with different tumor sizes. Scale bar: 1 mm. f) Microneedle patch administration. Scale bar: 1 mm. g) H&E staining of melanoma tissue and IHC staining of S100A1. Scale bar (H&E staining): 500 µm. Scale bar (IHC): 50 µm. h) IHC staining of S100A1 for tumors with different sizes. Scale bar: 50 µm.

To investigate the relationship between the S100A1 expression level in ISF and melanoma progression, five mice were injected with melanoma cells to induce melanoma. The microneedle patch extraction was conducted on different days to obtain different tumor sizes (**Figure** **6**a–e). The administration of microneedle has been shown in Figure 6f, in which the microneedle was fixed with a 3 m Tegaderm film for 30 min to ensure a sufficient extraction of ISF. The volume of extracted ISF was determined based on the weight difference before and after administration. After that, the extracted microneedle was transferred into a centrifuge tube containing 80 µL sample dilution buffer for elution overnight. The elution time has been optimized and there is no significant difference in the optical density for 30 min and overnight elution (Figure S4, Supporting Information). To compare to the conventional serum based S100A1 detection, the blood was collected from the same mouse after the microneedles administration and measured using ELISA. After the ISF extraction and blood sampling, the melanoma tissues (Figure 6a–e) were sliced for immunohistochemistry (IHC) staining, a standard method taken during clinical diagnosis. Based on the IHC staining of melanoma with distinct size differences (Figure 6g,h), however, it is hard to obtain the quantitative results of S100A1 concentration in different tumors.

### 2.6 ELISA and Microchip Detection of S100A1 Extracted from Animal Model

**Figure 7:**
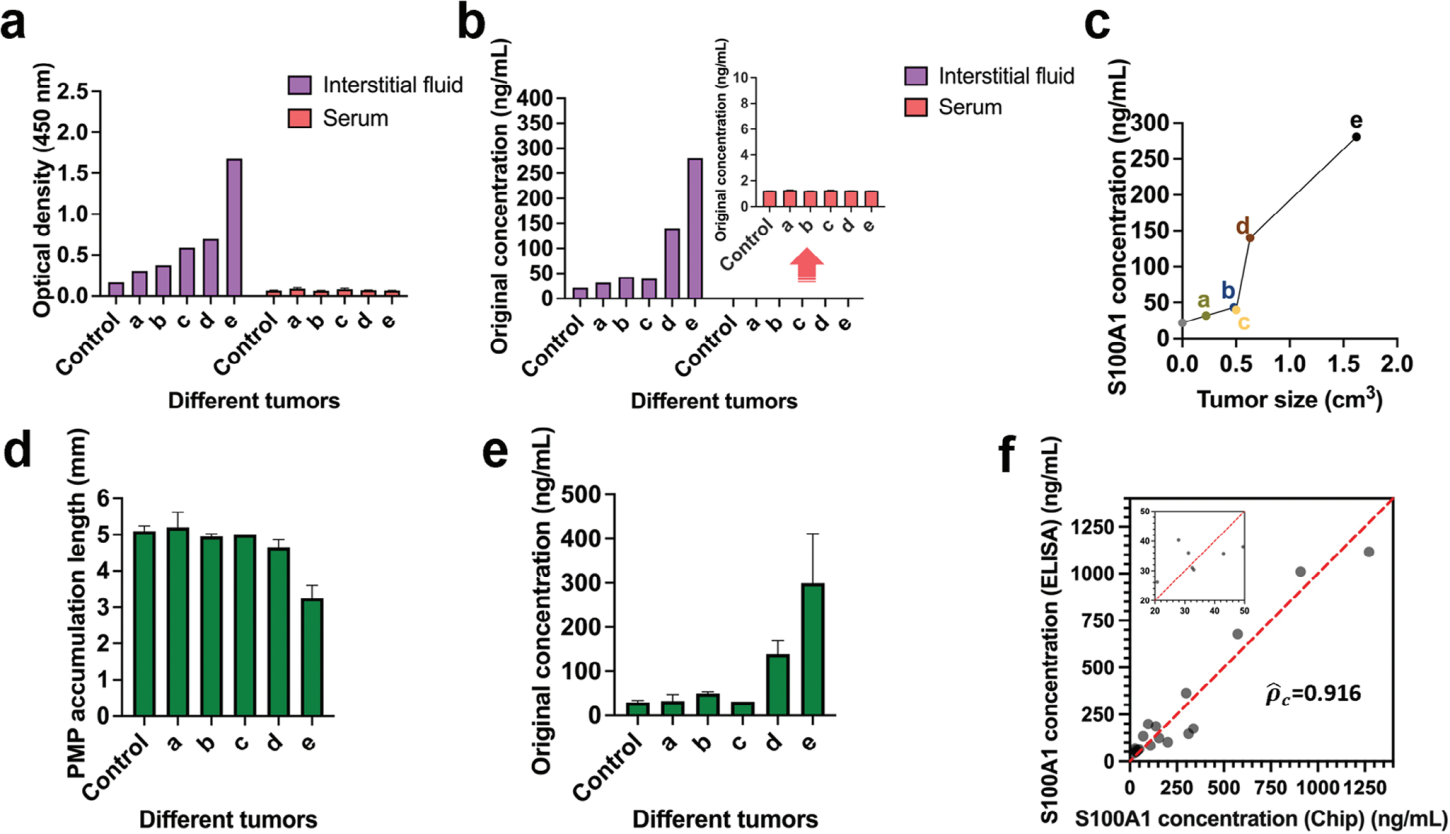
Quantitative measurement of S100A1 based on ELISA and microfluidic chip. a) ELISA measurement of S100A1 extracted from ISF and serum. b) Original concentration of S100A1 in ISF and serum based on the inverse regression. c) Relationship between the tumor size and S100A1 expression level. d) Microchip measurement of S100A1 extracted from ISF (n = 3). e) Original concentration of S100A1 in ISF based on the inverse regression (n = 3). f) Comparison of S100A1 expression levels measured by mouse S100A1 ELISA kit (y‐axis) and microfluidic chips (x‐axis). The correlation was determined on the basis of Lin's concordance correlation coefficient ${\hat{\rho }}_{c}$ = 0.916, validating a moderate agreement between the gold standard ELISA and the proposed microfluidic chip.

We next investigated the S100A1 concentration in tumors with different sizes based on on‐site swellable microneedle patch‐assisted sampling. The extracted ISF was first measured using the gold standard ELISA measurement. With the increased tumor sizes, the absorbance value of S100A1 in ISF exhibits a growing trend in ELISA (**Figure** **7**a). However, the trend is not observed in the serum sample, suggesting that serum S100A1 may not be sufficiently informative. Based on the inverse regression using the standard curve established above (Figure 4c), the original concentration of S100A1 was determined where the S100A1 concentration in ISF was significantly higher than that in serum (Figure 7b) and a relationship between the tumor size and the expression level of S100A1 was established (Figure 7c), demonstrating that the microneedle‐assisted on‐site S100A1 extraction is more effective than the serum‐based sampling. Next, the microchip based S100A1 detection in ISF was performed and a decreased PMP accumulation length was observed with the increase in tumor size (Figure 7d). After inverse regression, the concentration of S100A1 in different tumor sizes was determined (Figure 7e). In addition to the tumors with distinct size differences (a–e), fourteen mice with different tumor sizes were administrated using the microneedles, and the extracted ISF was measured using both ELISA and the microfluidic chip to determine the S100A1 concentration (Figure S5, Supporting Information). Finally, the result obtained from the microfluidic chip was compared with the result from the ELISA measurement. As shown in Figure 7f, a moderate correlation (${\hat{\rho }}_{c}$ = 0.916) was obtained based on Lin's concordance correlation coefficient (CCC) interpretation guideline, demonstrating that the microfluidic chip measurement is comparable with the conventional gold standard ELISA test for the visual quantification of S100A1.^[^ ^33^ ^]^

## 3 Conclusion

Melanoma is a skin cancer with a high mortality rate, making routine self‐monitoring particularly important. However, traditional screening methods such as dermoscopy and tissue biopsy require professional procedure and interpretation, which is impractical for routine inspection and diagnosis by the end‐users. Moreover, serum based S100 family protein detection is not sensitive due to blood dilution and is less specific since the circulating serum S100 biomarker is also related to other diseases. In this study, we developed an on‐site, minimally invasive sampling utilizing a swellable microneedle patch for extracting S100A1 from the interstitial fluid and direct visualization of the S100A1 level via a microfluidic particle dam. The hydrogel‐fabricated swellable microneedle with sufficient mechanical strength provides a simplified sampling method using normal thumb pressing and exhibits high biocompatibility for ISF extraction. On the other hand, a self‐powered microfluidic chip offers a user‐friendly interface for the determination of S100A1. Combining the merits of the miniaturized microneedle and microfluidic system, it has great potential for the routine self‐inspection of skin. In the future, UV exposure instead of the melanoma cell injection will be taken to better simulate the natural process of melanoma, to further prove the feasibility of our detection principle. Moreover, to ease the handling and sample analysis, a wearable platform for in situ melanoma diagnosis by integrating the microneedle patch with a microfluidic system will be explored.

## 4 Experimental Section

### Synthesis of Methacrylated Hyaluronic Acid (MeHA) and Fabrication of Microneedle Patch

The synthesis of methacrylated hyaluronic acid (MeHA) was based on the protocol reported before.^[^ ^28^, ^29^ ^]^ First, sodium hyaluronate, a sodium salt of hyaluronic acid (HA), was dissolved in water, and stirred evenly overnight. The methacrylic anhydride and N, N‐dimethylformamide were then added and the pH was adjusted to 8–9 with sodium hydroxide solution with stirring at 4° for 18 h. Sodium chloride was added to a concentration of 0.5 m before the precipitation of MeHA in ethanol and finally washed three times with ethanol and dissolved in deionized (DI) water. The purified product was lyophilized and characterized by ^1^H NMR spectroscopy. For the fabrication of cross‐linked MeHA microneedle patches, the synthesized MeHA (50 mg/mL) was mixed with a photoinitiator (Irgacure 2959, 0.5 mg mL^−1^) in DI water. With continuous stirring overnight, the mixture was poured onto a female PDMS mold demolded from a stainless‐steel master mold (300 µm base diameter, 5 µm tip radius, and 1000 µm height). To facilitate the filling up of cavities, the PDMS mold was centrifuged at 5000 rpm for 3 min to force material into needle voids. After that, a supplementary mixture solution was added to form a substrate of the microneedle patch and dried at room temperature in a fume hood overnight. After drying, the microneedle patch was obtained with careful detachment from the female PDMS mold, trimmed and irradiated with a UV lamp (wavelength = 360 nm, intensity = 17.0 mW cm^−2^, model 30, OAI) for 3 min before use.

### Immune Cell Staining

The cytotoxicity of the MeHA microneedles was first evaluated by the Live/Dead kit (Molecular Probes). The NIH/3T3 cell mouse fibroblasts (ATCC) were treated similarly with different microneedle soaking solutions that were then removed after 24 h incubation. Then, the cells were stained by ethidium homodimer‐1 (0.5 µm) and calcein AM (0.25 µm) according to the instructions of the Live/Dead kit. After staining, the samples were then observed under fluorescence microscope (Nikon). Furthermore, AlamarBlue cell viability assay (ThermoFisher) was also employed to measure the cytotoxicity of the MeHA microneedles. Briefly, NIH/3T3 cell mouse fibroblasts (ATCC) were first cultured in the Dulbecco's modified Eagle's medium containing 10% fetal bovine serum. The 10–100 kDa HA microneedle, 10–100 kDa MeHA microneedle, and 200–400 kDa MeHA microneedle were soaked in the abovementioned culture medium for 24 h. Then the cell culture medium was replaced by the soaking solution and was incubated for another 24 h. Later, the solution was removed and the cells were then washed once with PBS and incubated with complete media containing 10% AlamarBlue for 4 h at 37 °C. Finally, the absorbance of AlamarBlue at 570 nm was measured using a microplate reader (SpectraMax M5e).

### Skin Penetration Efficiency Test and Histology

The influence of microneedle penetration on the skin was then evaluated on mice models. The 10–100 kDa MeHA microneedles were applied on the backing area of the mice via a thumb press and were then peeled off after fully swelling. The optical images of the mice backing area were taken to observe the skin recovery 0, 1, 3, 5, and 15 min after penetration. Then the mice were sacrificed 30 min later and the backing area of the skin was harvested. The harvest mice's skin was then sliced by frozen section (Cryostar NX70, thermal fisher) and stained for histological analysis by haematoxylin and eosin (H&E) staining. The skin slices were observed by the Nikon Eclipse Ti‐E microscope.

### Preparation of Skin Model

A skin model was developed by preparing the agarose hydrogel (1.3 wt.%) covered with a film where the film layer represents the impermeable stratum corneum and epidermis, and the agarose hydrogel represents the upper dermis layer containing interstitial fluid. Fluorescein isothiocyanate (FITC) dextran (15 kDa) or S100A1 standards was dissolved in the skin model and extracted using the microneedles fabricated with different molecular weights (10–100 kDa and 200–400 kDa) of HA. The FITC‐dextran was prepared at 1 mg mL^−1^ as the final concentration in the skin model. For S100A1, 10 µL of S100A1 standards with different concentrations were mixed with 490 µL agarose gel (1.3 wt.%) and waited for 10 min for gelatin. The final S100A1 concentrations in the skin model were designed to be 0, 0.625, 1.25, 2.5, 5, 10, 20, 40, 100, and 200 ng mL^−1^. For the establishment of standard curves for both ELISA and microfluidic chip measurements, S100A1 with different concentrations ranging from 0 – 4,000 ng mL^−1^ were prepared in skin model and extracted using the microneedles with optimized molecular weight of HA.

### Extraction of FITC‐Dextran and S100A1 in Skin Model

After extraction of FITC‐Dextran, the microneedles were observed using fluorescence microscopy. For S100A1, the microneedles were administrated onto the skin model for 15 min followed by the elution overnight in 150 µL sample dilution buffer (20 mm Tris, 150 mm NaCl, 0.1% BSA and 0.05% Tween 20, pH 7.2–7.4, 0.2 µm filtered).

### Antibodies Conjugation of Microparticles

First, the carboxylated MMPs and PMPs were activated by 1‐ethyl‐3‐(3‐dimethylaminopropyl)carbodiimide/N‐hydroxysuccinimide (EDC/NHS). The MMPs and PMPs were first washed twice with 200 µL of activation buffer (0.1 m MES in DI water, pH 5.0), followed by the addition of 100 µL of EDC (50 mg mL^−1^) and 100 µL of NHS (50 mg mL^−1^) prepared in activation buffer. The EDC/NHS activation takes 15 min with continuous mixing at room temperature. After activation, the microparticles were washed three times in coupling buffer 1 (0.01 m PBS containing 0.2% Tween 20, pH 8.5) and adjusted to the original volume. Subsequently, the microparticles were mixed with the corresponding antibodies to be conjugated (MMPs‐Detection antibody (Det) and PMPs‐Capture antibody (Cap)) and incubated at 4 °C overnight with constant mixing. After that, the modified microparticles were washed with coupling buffer 1 and resuspended in 200 µL of Casein blocking buffer for 30 min, followed by the washing step with coupling buffer 2 (0.01 m PBS containing 1% Tween 20, pH 8.5).

### Flow Cytometry

Flow cytometry was employed to characterize the antibodies conjugation onto the microparticles and to preliminarily optimize the number of detection antibodies on MMPs and capture antibodies on PMPs. For MMP, 3.5 µL activated MMP (0.797 µm diameter, 1.725 × 10^11^ microspheres mL^−1^) and 2 µL different concentrations of detection antibody were gently mixed overnight in coupling buffer 1 at 4 °C. It was then washed three times using coupling buffer 1 and blocked as mentioned above. After that, it was reacted with 2 µL of anti‐human IgG antibody (2 mg ml^−1^) for 1 h at room temperature. Finally, the MMPs were washed three times again and adjusted to 500 µL using coupling buffer 1 for measurement. For PMPs, 3.5 µL activated PMP (15.3 µm in diameter, 5.146 × 10^7^ microspheres mL^−1^) and 2 µL capture antibody with different concentrations were mixed at 4 °C for overnight, followed by washing and blocking. Next, PMP immobilized with capture antibody was reacted with 2 µL of anti‐human IgG antibody (2 mg mL^−1^) for 1 h at room temperature, which was then washed and adjusted to 500 µL. For measurement, emissions were collected through allophycocyanin (APC) fluorescence channels, and 10 000 particles were analyzed for each sample. The average value was calculated to represent the fluorescence intensity.

### Microchip Fabrication

The microchip consisting of a Norland optical adhesive 63 (NOA 63, Norland Products, USA) layer and a Polydimethylsiloxane (PDMS) layer was fabricated based on the previous study.^[^ ^34^ ^]^ In brief, the fabrication of NOA 63 layer was based on soft‐lithography with a predesigned mask. SU‐8 2015 photoresist (Gersteltec Sarl, Switzerland) was first spin‐coated onto a silicon wafer (Suzhou Crystal Silicon Electronic & Technology Co., Ltd.) at 1200 rpm. As such, the thickness of the SU‐8 master is 25 ± 0.3 µm. After obtaining the SU‐8 master, PDMS precursor (elastomer base: curing agent = 10:1, SylgardTM 184, Dow Corning, USA) was poured onto the SU‐8 pattern and cured in an oven at 70° for 2 h. After curing, the PDMS with microchannel pattern (the first PDMS) was cut, peeled off, and then treated with oxygen plasma (Harrick Plasma, 400 mTorr) for 2 min before the gas‐phase deposition of trichloro(1H,1H,2H,2H‐perfluorooctyl) silane 97% (J&K Scientific Ltd.) for 6 h. Next, the PDMS precursor was poured onto the silane‐treated surface of the first PDMS to cast the second PDMS. The NOA 63 was then smeared on top of the second PDMS and covered by a polypropylene film (KOKUYO, Japan) with 100 µm thickness. Next, the NOA 63 was UV‐cured for 25 s and peeled off. On the other hand, the PDMS layer with a sample loading chamber (2 mm in height) was fabricated from a 3D printed mold that was prepared with ethanol washing, ultrasonic cleaning, post UV exposure (4 min), and hard baking (120 °C, 2 h) after 3D printing (Photon Mono SE, Anycubic) to avoid PDMS curing inhibition. PDMS precursor (elastomer base: curing agent = 10:1) was then cast onto the 3D printed mold and demolded, followed by the treatment of lubricant to make the sample loading chamber hydrophilic. After treatment and removal of lubricant residual, the PDMS was bonded with the NOA 63 layer after plasma treatment (Harrick Plasma, 800 mTorr, 2 min). After that, a neodymium magnet with a size of 2.6 mm × 1.8 mm × 1.5 mm was glued at the bottom of the magnetic separator with a 1 mm gap in the NOA 63 layer.

### Detection on the Microchip

For on‐chip detection of S100A1 standards, 5 µL of PMPs conjugated with the capture antibody (PMPs‐Cap), 5 µL of MMPs conjugated with the detection antibody (MMPs‐Det), and 5 µL of S100A1 standards with different concentrations were gently mixed for 30 min at room temperature (total volume: 15 µL). After that, three microliters of the mixture were loaded into the sample loading chamber of a microfluidic chip for measurement. For the detection of S100A1 extracted from the skin model using a microneedle patch, 5 µL of the eluted samples from the microneedle patch were first reacted with MMPs‐Det for 30 min. After that, MMPs were washed three times with coupling buffer 1 and adjusted the volume to 10 µL, followed by the addition of 5 µL of PMPs‐Cap for another 30 min reaction with continuous shaking. Finally, 3 µL of the solution above was loaded into the microchip for measurement. For the detection of S100A1 extracted from the mice using a microneedle patch, the protocol is the same as that for S100A1 extracted from the skin model by replacing the 5 µL of the eluted samples extracted from the skin model with the samples extracted from the mice.

### Enzyme‐Linked Immunosorbent Assay

An ELISA kit was used based on the protocol provided by the manufacturer (Sino Biological, Inc. Beijing, China). For the proof‐of‐concept of S100A1 extraction from the skin model, 100 µL of sample from 150 µL total volume after elution was added to the microplate's pre‐coated wells. While for the establishment of the ELISA standard curve of S100A1 extracted using a microneedle patch, the eluted sample from microneedles was diluted 30 times (5 µL eluted sample in 150 µL sample dilution buffer) and 100 µL of diluted samples were added into the microplate wells. For mouse serum detection, the serum was diluted 30 times first and 100 µL of diluted samples were added into the microwells for measurement. For the mouse ISF detection, the eluted ISF from microneedles was diluted 30 times and 100 µL of diluted samples were added into the microwells for measurement. After the sample was added and reacted for 2 h, the microplate was washed with washing buffer three times, and the detection antibody with HRP conjugation was added and reacted for 1 h. After finishing another washing cycle, 200 µL of TMB substrate solution was added to each well and incubated at room temperature in the dark for 15 min before adding 50 µL of stop solution to each well and measured using a microplate reader (SpectraMax M5e Multi‐Mode Microplate Reader, Molecular Devices) at 450 nm after the reaction stopped.

### Animal Experiment

To induce melanoma in mice, 3 × 10^6^ B16F10 cells (ATCC‐CRL‐6475, American Type Culture Collection, Manassas, VA, USA) were used. The cell was cultured in Dulbecco's Modified Eagle Medium (4 mm L‐glutamine, 4500 mg L^−1^ glucose, 1 mm sodium pyruvate and 3.7 g L^−1^ sodium bicarbonate, Life Technologies) supplemented with 10% fetal bovine serum and 1% penicillin–streptomycin (Life Technologies). The cells were suspended in 200 µL of phosphate‐buffered saline and injected subcutaneously into the dorsal side of the two legs of each mouse. Six 1‐month‐old female Nude balb/c mice were used in this study and one of them was regarded as the control group and others were injected with B16F10 cells to induce melanoma. The animals were housed under temperatures ranging between 22.5 and 24.5 °C with a 12:12 h light‐dark cycle and received ad libitum standard mice chow and water. The protocol was approved by the Committee on the Ethics of Animal Experiments of the Laboratory Animal Research Unit of the City University of Hong Kong (Permit Number: A‐0667) and conducted in accordance with the Guide for the Care and Use of Laboratory Animals.

### H&E Staining and Immunohistochemistry (IHC) for Sliced Melanoma Tissue

The melanoma tissue was harvested and fixed in 4% PFA and dehydrated with 30% sucrose. Tumors were embedded in an optimal cutting temperature (OCT) compound for sectioning. For immunohistochemical staining, the samples were air‐dried at room temperature overnight. Tissue sections were washed with Phosphate‐Buffered Saline, 0.1% Tween (PBST) buffer, and blocked with 5% goat serum and 2.5% BSA for 2 h. The sections were incubated in 1:500 anti‐S100A1 antibody (Sino Biological, Inc. Beijing, China) in 5% BSA overnight. After washing, the sections were incubated with horseradish peroxidase‐coupled goat anti‐rabbit secondary antibody (Cell Signaling) for an hour in the blocking buffer. The Pierce DAB Substrate Kit with 3,3‐diaminobenzidine (Thermo Scientific) was used for detection. Tissue sections were then rinsed in PBST buffer and the nucleus was counterstained with hematoxylin. Finally, the sections were dehydrated and mounted.

### Statistical Analysis

All statistical analyses were performed in a blind manner using GraphPad Prism 9 software. All results were represented as mean ± standard deviation. The statistical details can be found in Equations S1–S9 (Supporting Information).

## Conflict of Interest

The authors declare no conflict of interest.

## Supporting information

Supporting Information

## Data Availability

The data that support the findings of this study are available from the corresponding author upon reasonable request.

## References

[1] M. Arnold , D. Singh , M. Laversanne , J. Vignat , S. Vaccarella , F. Meheus , A. E. Cust , E. de Vries , D. C. Whiteman , F. Bray , Jama Dermatol. 2022, 158, 495. 35353115 10.1001/jamadermatol.2022.0160 PMC8968696

[2] L. E. Davis , S. C. Shalin , A. J. Tackett , Cancer Biol. Ther. 2019, 20, 1366. 31366280 10.1080/15384047.2019.1640032 PMC6804807

[3] H. Mishra , P. K. Mishra , A. Ekielski , M. Jaggi , Z. Iqbal , S. Talegaonkar , J. Cancer Res. Clin. 2018, 144, 2283. 10.1007/s00432-018-2726-1 PMC11813321 30094536

[4] G. A. Holmes , J. M. Vassantachart , B. A. Limone , M. Zumwalt , J. Hirokane , S. E. Jacob , Fed. Pract. 2018, 35, S39. 30766399 PMC6375419

[5] A. T. Young , N. B. Vora , J. Cortez , A. Tam , Y. Yeniay , L. Afifi , D. Yan , A. Nosrati , A. Wong , A. Johal , M. L. Wei , Pigm. Cell Melanoma Res. 2021, 34, 288. 10.1111/pcmr.12907 32558281

[6] E. Saliba , J. Bhawan , Dermatopathology 2021, 8, 359. 34449584 10.3390/dermatopathology8030040 PMC8395931

[7] R. Vaidyanathan , R. H. Soon , P. Zhang , K. Jiang , C. T. Lim , Lab Chip 2019, 19, 11. 10.1039/c8lc00684a 30480287

[8] A. Eisenstein , E. C. Gonzalez , R. Raghunathan , X. X. Xu , M. Z. Wu , E. O. McLean , J. McGee , B. Ryu , R. M. Alani , Mol. Diagn. Ther. 2018, 22, 203. 29411301 10.1007/s40291-018-0318-z

[9] N. T. Wright , K. M. Varney , K. C. Ellis , J. Markowitz , R. K. Gitti , D. B. Zimmer , D. J. Weber , J. Mol. Biol. 2005, 353, 410. 16169012 10.1016/j.jmb.2005.08.027

[10] T. Hoch , D. Schulz , N. Eling , J. M. Gómez , M. P. Levesque , B. Bodenmiller , Sci. Immunol. 2022, 7, eabk1692. 35363540 10.1126/sciimmunol.abk1692

[11] M. Hessler , E. Jalilian , Q. Y. Xu , S. Reddy , L. Horton , K. Elkin , R. Manwar , M. Tsoukas , D. Mehregan , K. Avanaki , Int. J. Mol. Sci. 2020, 21, 9583. 33339193 10.3390/ijms21249583 PMC7765677

[12] T. F. Xiong , F. Q. Pan , D. Li , Melanoma Res. 2019, 29, 23. 30216200 10.1097/CMR.0000000000000512 PMC6310472

[13] Y. Zhou , Y. W. Zha , Y. Q. Yang , T. Ma , H. L. Li , J. Y. Liang , Mol. Med. 2023, 29, 68. 37217870 10.1186/s10020-023-00662-1 PMC10204201

[14] G. Wang , H. N. Li , X. Q. Cui , T. Xu , M. L. Dong , S. Y. Li , X. R. Li , J. Cancer 2021, 12, 5760. 34475990 10.7150/jca.51855 PMC8408122

[15] S. Z. Zhang , Z. Wang , W. W. Liu , R. Lei , J. L. Shan , L. Li , X. C. Wang , Sci. Rep. 2017, 7, 39786. 28051137 10.1038/srep39786 PMC5209742

[16] T. Tian , X. K. Li , Z. Hua , J. L. Ma , Z. H. Liu , H. Y. Chen , Z. M. Cui , Discov. Med. 2017, 23, 235. 28595036

[17] B. Q. Tran , P. R. Miller , R. M. Taylor , G. Boyd , P. M. Mach , C. N. Rosenzweig , J. T. Baca , R. Polsky , T. Glaros , J. Proteome Res. 2018, 17, 479. 29172549 10.1021/acs.jproteome.7b00642

[18] Z. Y. Wang , J. Y. Luan , A. Seth , L. Liu , M. L. You , P. Gupta , P. Rathi , Y. X. Wang , S. S. Cao , Q. S. Jiang , X. Zhang , R. Gupta , Q. J. Zhou , J. J. Morrissey , E. L. Scheller , J. S. Rudra , S. Singamaneni , Nat. Biomed. Eng. 2021, 5, 64. 33483710 10.1038/s41551-020-00672-y PMC8020465

[19] P. P. Samant , M. M. Niedzwiecki , N. Raviele , V. Tran , J. Mena‐Lapaix , D. I. Walker , E. I. Felner , D. P. Jones , G. W. Miller , M. R. Prausnitz , Sci. Transl. Med. 2020, 12, eaaw0285. 33239384 10.1126/scitranslmed.aaw0285 PMC7871333

[20] R. Y. He , Y. Niu , Z. D. Li , A. Y. Li , H. Yang , F. Xu , F. Li , Adv. Healthcare Mater. 2020, 9, 1901201. 10.1002/adhm.201901201 31957291

[21] J. X. Wang , Z. Y. Lu , R. S. Cai , H. Q. Zheng , J. C. Yu , Y. Q. Zhang , Z. Gu , Lab Chip 2023, 23, 869. 36629050 10.1039/d2lc00790h

[22] S. W. Chen , Y. Niu , J. C. Yeo , Y. C. Liu , J. M. Qi , S. C. Fan , X. Y. Liu , J. Y. Lee , C. T. Lim , Nat. Rev. Bioeng. 2023, 1, 950.

[23] Z. Z. Xie , G. P. Chen , J. Y. Che , D. G. Zhang , Eng. Regener. 2022, 3, 420.

[24] H. X. Chen , F. K. Bian , Y. J. Zhao , Smart Med. 2022, 1, e20220001. 10.1002/SMMD.20220001 PMC11235995 39188737

[25] H. Y. Yang , X. Jiang , Y. N. Zeng , W. Zhang , Q. Q. Yuan , M. R. Yin , G. S. Wu , W. Li , Chem. Eng. J. 2023, 455, 140730.

[26] X. Q. Huang , L. Z. Chen , T. J. Sha , Y. J. Lin , R. M. Zeng , J. Xu , S. Z. Chen , H. H. Cai , J. L. Zhang , H. B. Zhou , P. H. Sun , X. Y. Jiang , ACS Nano 2023, 17, 20073. 37792448 10.1021/acsnano.3c05638

[27] S. Totti , K. W. Ng , L. Dale , G. P. Lian , T. Chen , E. G. Velliou , Sens. Actuators B: Chem. 2019, 296, 126652.

[28] M. J. Zheng , Y. Y. Zhang , T. L. Hu , C. J. Xu , Bioeng. Transl. Med. 2023, 8, e10413. 37693058 10.1002/btm2.10413 PMC10487322

[29] H. Chang , M. J. Zheng , X. J. Yu , A. Than , R. Z. Seeni , R. J. Kang , J. Q. Tian , D. P. Khanh , L. B. Liu , P. Chen , C. J. Xu , Adv. Mater. 2017, 29, 1702243. 10.1002/adma.201702243 28714117

[30] M. Ahearne , Y. Yang , A. J. El Haj , K. Y. Then , K. K. Liu , J. R. Soc. Interface 2005, 2, 455. 16849205 10.1098/rsif.2005.0065 PMC1618501

[31] Z. B. Guo , K. Hu , J. F. Sun , T. Z. Zhang , Q. Y. Zhang , L. Song , X. Z. Zhang , N. Gu , ACS Appl. Mater. Interfaces 2014, 6, 10963. 24989081 10.1021/am5023946

[32] Z. C. Zhao , Y. Y. Bao , L. T. Chu , J. K. L. Ho , C. C. Chieng , T. H. Chen , Lab Chip 2017, 17, 3240. 28869261 10.1039/c7lc00836h

[33] J. J. Liao , J. W. Lewis , PDA J. Pharm. Sci. Technol. 2000, 54, 23. 10778304

[34] M. H. Wu , S. Y. Wu , G. B. Wang , W. G. Liu , L. T. Chu , T. Y. Jiang , H. K. Kwong , H. L. Chow , I. W. S. Li , T. H. Chen , Sci. Adv. 2022, 8, eabn6064. 35658040 10.1126/sciadv.abn6064 PMC9166397

