# Supplementary material for: On‐Site Melanoma Diagnosis Utilizing a Swellable Microneedle‐Assisted Skin Interstitial Fluid Sampling and a Microfluidic Particle Dam for Visual Quantification of S100A1
Source: Adv Sci (Weinh). 2024 Feb 28;11(16):2306188. doi: 10.1002/advs.202306188 (PMC11040363; doi:10.1002/advs.202306188)
Supplement: Supplementary file 1 — Supporting Information [file ADVS-11-2306188-s001.pdf]

ADVANCED SCIENCE
Open Access

Supporting Information

for *Adv. Sci.*, DOI 10.1002/advs.202306188

On-Site Melanoma Diagnosis Utilizing a Swellable Microneedle-Assisted Skin Interstitial Fluid Sampling and a Microfluidic Particle Dam for Visual Quantification of S100A1

Gaobo Wang, Yuyue Zhang, Hoi Kwan Kwong, Mengjia Zheng, Jianpeng Wu, Chenyu Cui, Kannie W. Y. Chan, Chenjie Xu and Ting-Hsuan Chen**

Supporting Information

On-site Melanoma Diagnosis Utilizing a Swellable Microneedle-assisted Skin Interstitial Fluid Sampling and a Microfluidic Particle Dam for Visual Quantification of S100A1

GaoboWang[1], *Yuyue Zhang*[1], *Hoi Kwan Kwong*[1], *Mengjia Zheng*[1], *Jianpeng Wu*[1], *Chenyu Cui*[1, 3], *Kannie W. Y. Chan*[1, 3], *Chenjie Xu*[1, *], *Ting-Hsuan Chen*[1-3, *]

[1] Department of Biomedical Engineering, City University of Hong Kong, 83 Tat Chee Avenue, Kowloon Tong, Hong Kong SAR, 999077, China

[2] City University of Hong Kong Shenzhen Research Institute, 8 Yuexing 1st Road, Shenzhen Hi-Tech Industrial Park, Nanshan District, Shenzhen, 518057, China

[3] Hong Kong Centre for Cerebro-Cardiovascular Health Engineering, Rm 1115-1119, Building 19W, 19 Science Park West Avenue, Hong Kong SAR, 999077, China

*Corresponding author
Email: chenjie.xu@cityu.edu.hk; thchen@cityu.edu.hk

G.W., Y.Z., and H.K.K. contributed equally to this work.

This file includes **Figure S1** to **S5** and **Equations S1** to **S9**.

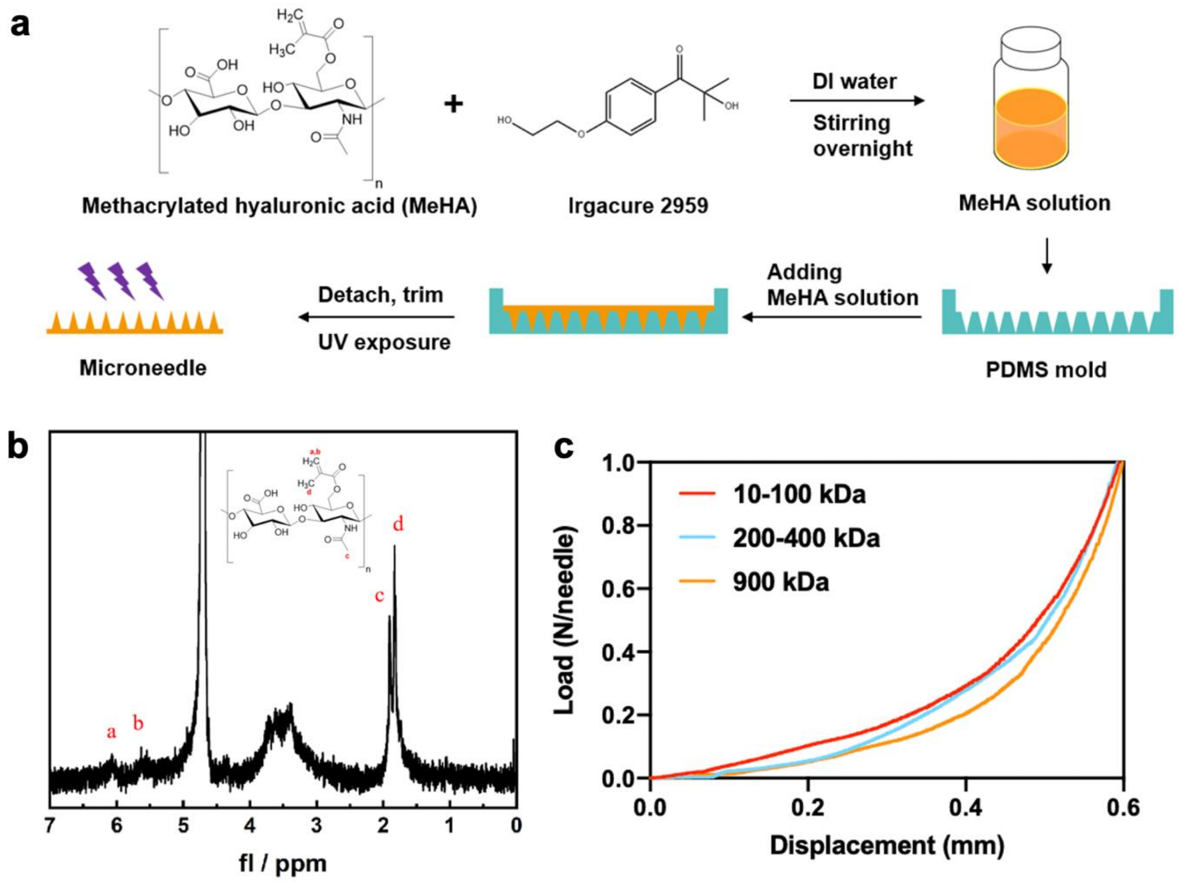

Figure S1. Fabrication of MeHA microneedles. (a) The fabrication procedure of the swellable microneedles. (b) ^{1}H NMR spectra of MeHA. The degree of modification was determined by digital integration of the anomeric protons signals or methyl protons signals of HA and of the methacrylate proton signals at ~6.1, ∼5.7, and ~1.9 ppm. (c) Compression test of MNs with different molecular weights (10-100 kDa, 200-400 kDa, 900 kDa).

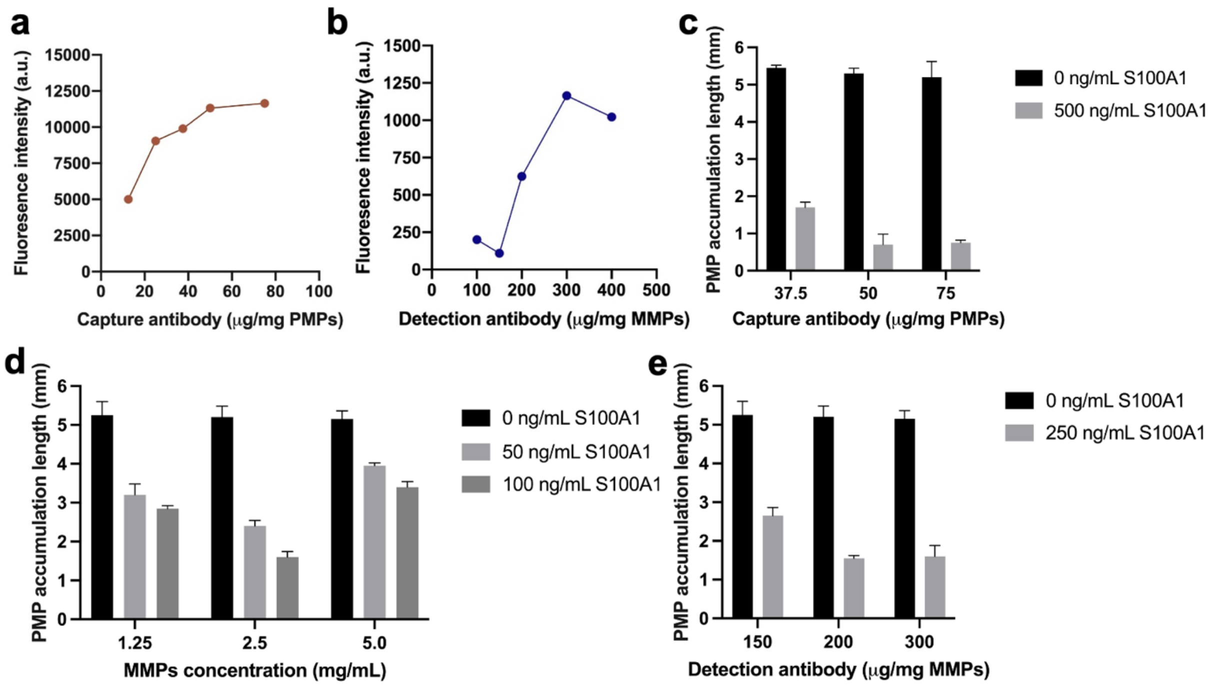

Figure S2. Optimization for antibody conjugation and particle concentration. (a-b) Preliminary optimization of antibodies amount conjugated onto microparticles using flow cytometry. (c) Optimization of capture antibody amount conjugated onto PMPs using microchip (5 mg/mL MMPs with 300 μg detection antibody per mg MMPs). (d) Optimization of the number of MMPs using microchip (20 mg/mL PMPs with 50 μg capture antibody per mg PMPs and 300 μg detection antibody per mg MMPs). (e) Optimization of detection antibody amount conjugated onto MMPs using microchip (2.5 mg/mL MMPs and 20 mg/mL PMPs with 50 μg capture antibody per mg PMPs).

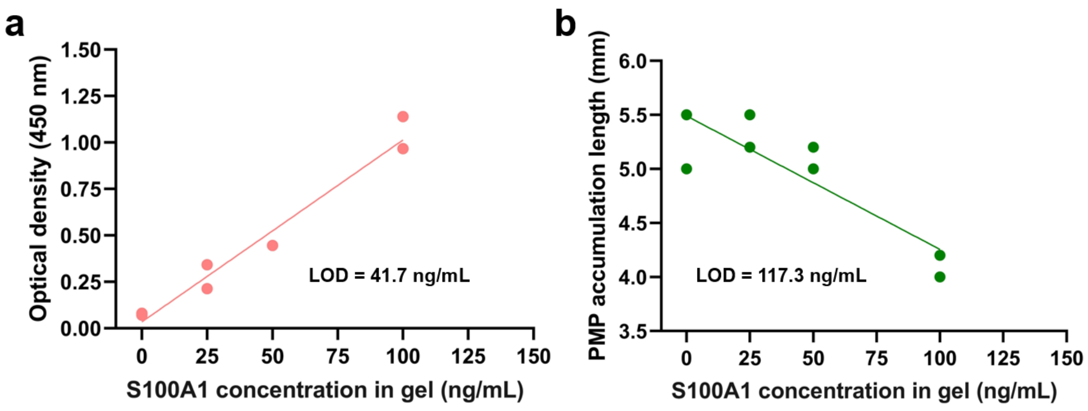

Figure S3. Limit of detection of ELISA measurement (a) and microfluidic chip measurement (b) for microneedles extracted S100A1 using skin model (Corresponding to Fig. 4b and Fig. 4d).

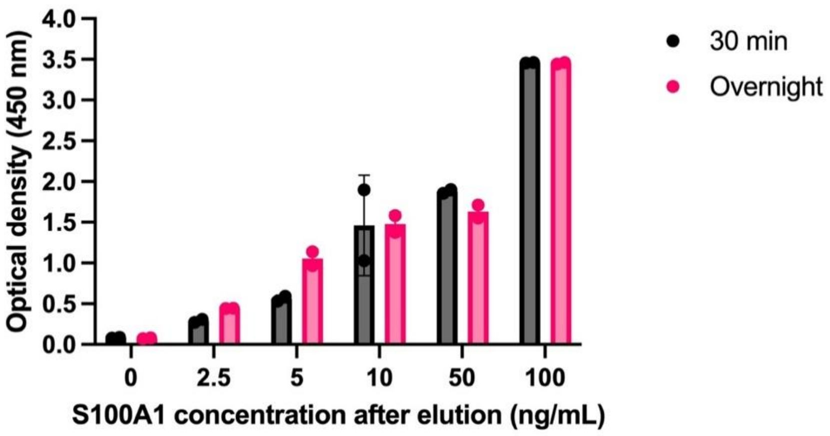

Figure S4. Optimization of elution time using the skin model. The S100A concentration after elution was defined based on the extracted volumes to ensure 20 times dilution.[1]

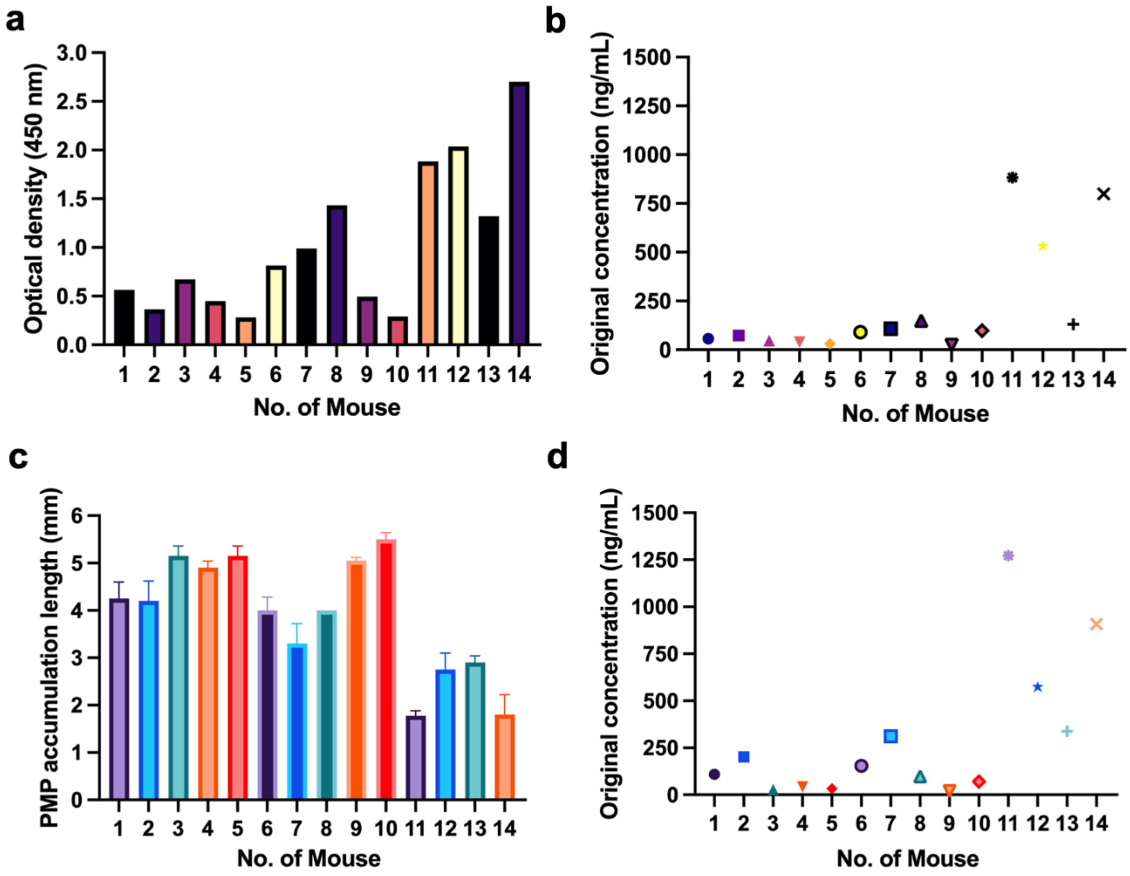

Figure S5. ELISA and microchip measurement of fourteen mice. (a) The optical density of S100A1 concentration extracted from fourteen mice. (b) Calculated the original concentration of S100A1 based on the inverse regression using the established ELISA standard curve (Figure 4c). (c) Microchip measurement of S100A1 extracted from fourteen mice and displayed with PMP accumulation lengths. (d) Calculated the original concentration of S100A1 based on the inverse regression using the microchip standard curve (Figure 4e).

Statistical analysis

1. Linear calibration curve

The calibration model is assumed to be $y = b_0 + b_1x$, using least-squares regression by incorporating the variance of model parameters, i.e. intercept b_0 and slope b_1, as shown in follows:

$$b_0 = \bar{y} - b_1\bar{x} \qquad \text{(S1)}$$

$$b_1 = \frac{\sum_{i=1}^{n}(x_i - \bar{x})y_i}{\sum_{i=1}^{n}(x_i - \bar{x})^2} \qquad \text{(S2)}$$

$$s_{y/x}^2 = \frac{\sum_{i=1}^{n}(y_i - y_i)^2}{n-2} \qquad \text{(S3)}$$

$$s_{b_0}^2 = s_{y/x}^2 \left(\frac{1}{n} + \frac{\overline{x}^2}{\sum_{i=1}^{n}(x_i - \overline{x})^2} \right) \qquad \text{(S4)}$$

$$s_{b_1}^2 = \frac{s_{y/x}^2}{\sum_{i=1}^{n}(x_i - \overline{x})^2} \qquad \text{(S5)}$$

where, $s_{b_0}^2$ and $s_{b_1}^2$ are the variances of *y* values, intercept b_0 and slope b_1, *n* is the total data point number, i.e. $n = \sum_{j=1}^{k} m_j$, *k* is the number of concentration levels, m_j is the number of replicates at the concentration level, and, $\overline{x} = \sum_{i=1}^{n} \frac{x_i}{n}$ and $\overline{y} = \sum_{i=1}^{n} \frac{y_i}{n}$. $y_i = b_0 + b_1 x_i$, defining the anticipated value of y for a specific concentration level.

Therefore, the calibration model can be written as:

$$\overline{y}_m^{\pm} = b_0 + b_1 x \pm t_{(1-\alpha/2,n-2)} s_{y/x} \left(\frac{1}{m} + \frac{1}{n} + \frac{(x-\overline{x})^2}{\sum_{i=1}^{n}(x_i - \overline{x})^2} \right)^{1/2} \qquad \text{(S6)}$$

where, $t_{(1-\alpha/2,\, n-2)}$ is the critical value of student *t* distribution where 1 - $\alpha/2$ is the confidence interval of two-tailed hypothesis tests, and $1/m$ is the contribution of the uncertainty from the average of m replicates in future observations.[2-3]

2. Limit of detection

The limit of detection can be obtained based on the non-central t-distribution:

$$x_D = \delta_{(\alpha,\beta,n-2)} \frac{s_{y/x}}{b_1} \left(1 + \frac{1}{n} + \frac{\bar{x}^2}{\sum_{i=1}^{n}(x_i - \bar{x})^2} \right)^{1/2} + x_{min} \qquad \text{(S7)}$$

where $x_{\min}$ is the minimum of *x* and $\delta_{(\alpha,\beta,n-2)}$ is the non-centrality parameter which protects against type I and type II errors (less than 5% of false positive/negative rate).[2]

3. Inverse regression

Inverse regression is utilized to get the estimated concentration based on the calibration model after reading the average distances. The equations are as follows:

$$\overset{\pm}{x_0} = \hat{x}_0 \pm t_{(1-\alpha/2,n-2)} s_{\hat{x}_0} \qquad \text{(S8)}$$

where, $\hat{x}_0$ is the predicted concentration, i.e. $\hat{x}_0 = (\bar{y}_{0m} - b_0)/b_1$, $\bar{y}_{0m}$ is the average of *m* replicated measurements and the variances of $\hat{x}_0$ can be derived as:

$$s_{\hat{x}_0}^2 = \frac{s_{y/x}^2}{b_1^2}\left(\frac{1}{m} + \frac{1}{n} + \frac{(\hat{x}_0 - \bar{x})^2}{\sum_{i=1}^{n}(x_i - \bar{x})^2}\right) \qquad \text{(S9)}$$

Hence, the error can be estimated as $t_{(1-\alpha/2,n-2)} s_{\hat{x}_0}$, where m = 3, and *t* = 1.645 for 95% of the confidence interval of two-tailed hypothesis.

Reference

[1] H. Chang, M. J. Zheng, X. J. Yu, A. Than, R. Z. Seeni, R. J. Kang, J. Q. Tian, D. P. Khanh, L. B. Liu, P. Chen, C. J. Xu, *Adv. Mater.* **2017**, *29*, 1702243.

[2] I. Lavagnini, F. Magno, *Mass Spectrom. Rev.* **2007**, *26*, 1-18.

[3] C. A. Clayton, J. W. Hines, P. D. Elkins, *Anal. Chem.* **1987**, *59*, 2506-2514.
